# Integrated multi‐omics analysis reveals drought stress response mechanism in chickpea (*Cicer arietinum* L.)

**DOI:** 10.1002/tpg2.20337

**Published:** 2023-05-10

**Authors:** Himabindu Kudapa, Arindam Ghatak, Rutwik Barmukh, Palak Chaturvedi, Aamir Khan, Sandip Kale, Lena Fragner, Annapurna Chitikineni, Wolfram Weckwerth, Rajeev K. Varshney

**Affiliations:** ^1^ Center of Excellence in Genomics & Systems Biology International Crops Research Institute for the Semi‐Arid Tropics (ICRISAT) Hyderabad India; ^2^ Molecular Systems Biology Lab (MOSYS), Department of Functional and Evolutionary Ecology University of Vienna Vienna Austria; ^3^ The Leibniz‐Institute of Plant Genetics and Crop Plant Research (IPK) Gatersleben Germany; ^4^ Vienna Metabolomics Centre (VIME) University of Vienna Vienna Austria; ^5^ Centre for Crop & Food Innovation, WA State Agricultural Biotechnology Centre, Food Futures Institute Murdoch University Murdoch Western Australia Australia

## Abstract

Drought is one of the major constraints limiting chickpea productivity. To unravel complex mechanisms regulating drought response in chickpea, we generated transcriptomics, proteomics, and metabolomics datasets from root tissues of four contrasting drought‐responsive chickpea genotypes: ICC 4958, JG 11, and JG 11+ (drought‐tolerant), and ICC 1882 (drought‐sensitive) under control and drought stress conditions. Integration of transcriptomics and proteomics data identified enriched hub proteins encoding isoflavone 4′‐O‐methyltransferase, UDP‐d‐glucose/UDP‐d‐galactose 4‐epimerase, and delta‐1‐pyrroline‐5‐carboxylate synthetase. These proteins highlighted the involvement of pathways such as antibiotic biosynthesis, galactose metabolism, and isoflavonoid biosynthesis in activating drought stress response mechanisms. Subsequently, the integration of metabolomics data identified six metabolites (fructose, galactose, glucose, myoinositol, galactinol, and raffinose) that showed a significant correlation with galactose metabolism. Integration of root‐omics data also revealed some key candidate genes underlying the drought‐responsive *“QTL‐hotspot”* region. These results provided key insights into complex molecular mechanisms underlying drought stress response in chickpea.

## Core Ideas

- Multi‐omics analysis of chickpea roots revealed complex molecular mechanisms underpinning drought stress response.
- Integration of transcriptome and proteome data uncovered hub proteins involved in drought stress response pathways.
- Metabolomic profiling identified six metabolites showing a significant correlation with galactose metabolism.
- Transcriptome‐proteome integration revealed prominent differential expression of key genes underlying the *“QTL‐hotspot”* region.

**Abbreviations**
ABA: abscisic acid
CID: collision‐induced dissociation
DEG: differentially expressed gene
DEP: differentially expressed protein
FDR: false discovery rate
FPKM: fragments per kilobase of transcript per million mapped reads
GC‐TOF‐MS: gas chromatography coupled to time‐of‐flight mass spectrometry
GO: gene ontology
ICRISAT: International Crops Research Institute for the Semi‐Arid Tropics
IL: introgression line
KEGG: Kyoto Encyclopedia of Genes and Genomes
LEA: late embryogenesis abundant
LRR: leucine‐rich repeat
LRX: leucine‐rich repeat extensin
MABC: marker‐assisted backcrossing
NSAF: normalized spectral abundance factor
PC: principal component
QTL: quantitative trait locus
ROS: reactive oxygen species
TMS: trimethylsilyl

## 1 INTRODUCTION

Chickpea (*Cicer arietinum* L.) is an important food legume cultivated over an area of ∼14.84 million hectares with a productivity of ∼15.08 million metric tons (FAOSTAT, 2020). It is mainly grown in arid and semiarid tropics, and it suffers substantial yield loss due to abiotic stresses such as drought, salinity, and heat. The increasing environmental fluctuations and the complex nature of drought are among the major factors limiting chickpea yields, often leading to 60%–70% annual yield losses (Barmukh, Roorkiwal, Garg, et al., 2022; Hajjarpoor et al., 2018). Genetic improvement and development of genetically superior germplasm is the most sustainable way to reduce the effect of drought stress (Varshney, Barmukh, et al., 2021). In this direction, deployment of one or combination of more than one of the following approaches—drought escape, drought avoidance, and drought tolerance—is expected to deliver better crop varieties with enhanced drought stress adaptation.

In the case of chickpea, although conventional breeding has delivered several drought‐tolerant varieties, the rate of genetic gain for yield under drought stress conditions needs to be accelerated. In several of our earlier studies, we have demonstrated that the prolific root system (mainly through drought avoidance mechanism) contributes to grain yield under drought stress conditions in chickpea (Barmukh, Roorkiwal, Garg, et al., 2022; Kashiwagi et al., 2015). Therefore, to complement conventional breeding efforts, understanding of drought stress response at molecular level in chickpea roots is necessary for the improvement of elite varieties through molecular breeding. To this end, efforts such as quantitative trait locus (QTL) mapping (Kale et al., 2015; Kushwah et al., 2022; Thudi et al., 2014; Varshney et al., 2014), candidate gene‐based allele diversity analysis (Roorkiwal et al., 2014), genome‐wide association study (GWAS) (Thudi et al., 2014), and transcriptome profiling (Kudapa et al., 2014, 2018; Varshney et al., 2009) have been undertaken to identify QTLs/genes associated with root traits. For instance, a *“QTL‐hotspot”* region harboring 13 robust QTLs for 12 drought stress response‐related traits (including root traits) and explaining up to 58.20% phenotypic variation was identified in an intraspecific mapping population (ICC 4958 × ICC 1882) (Varshney et al., 2014). Introgression of this “*QTL‐hotspot*” region from the donor parent ICC 4958 into an elite variety (JG 11) using marker‐assisted backcrossing (MABC) approach improved root‐related traits and drought stress response in chickpea (Varshney, Gaur, et al., 2013). Several introgression lines (ILs) containing the “*QTL‐hotspot*” region showed better phenotypic performance compared to their recurrent parent (Barmukh, Roorkiwal, Dixit, et al., 2022; Bharadwaj et al., 2021). While these different/independent studies provided informative cues about drought stress response, the molecular mechanisms in chickpea roots conferring drought tolerance are not yet well understood.

With an objective to understand the molecular basis of drought stress response in chickpea, this study undertakes a systemic and integrative “multi‐omics” approach to demonstrate the differential accumulation of transcripts, proteins, and metabolites in root tissues under drought conditions using four chickpea genotypes contrasting for drought tolerance, viz., ICC 4958 (drought‐tolerant) and JG 11 (drought‐tolerant), JG 11+ (IL, drought‐tolerant), and ICC 1882 (drought‐sensitive). These genotypes represent parents of two intraspecific mapping populations segregating for root traits, ICC 4958 × ICC 1882 and ICC 4958 × JG 11, whereas JG 11+ is an IL derived from ICC 4958 × JG 11. While the “*QTL‐hotspot*” region was identified in ICC 4958 × ICC 1882 population, it was transferred into an elite and leading chickpea variety JG 11 by crossing with ICC 4958. This study provides an integrated view of drought stress response, extending from transcript to protein and metabolite expression. Besides, it also offers novel insights into the molecular processes involved and emphasizes the importance of a systems biology approach to uncover regulatory mechanisms underpinning complex traits such as drought.

## 2 MATERIALS AND METHODS

### 2.1 Plant material and stress treatments

Four chickpea genotypes with contrasting responses to drought stress, viz., ICC 4958 (drought‐tolerant), JG 11 (drought‐tolerant), an IL JG 11+ (drought‐tolerant), and ICC 1882 (drought‐sensitive), were selected for transcriptome, proteome, and metabolome analyses (Figure S1). The experiment was set up under controlled glasshouse conditions at the International Crops Research Institute for the Semi‐Arid Tropics (ICRISAT), Patancheru, India. A total of three biological replications were maintained for each genotype under both control and stress conditions.

The plants were subjected to progressive drought stress at the flowering stage under glasshouse conditions (day/night temperature: 32/25°C; relative humidity: 40%–80%) as described in Varshney et al. (2009). In brief, drought stress treatment was imposed when the plants reached about 25 days old seedling stage, with the pots completely saturated with water and allowed to drain overnight. The next morning, the top soil layer was covered with a plastic sheet, and a thin layer of low‐density polyethylene granules was added to prevent soil evaporation. The plants grown under control treatment were maintained at ∼80% field capacity for the entire experiment duration. By contrast, the other set of plants was subjected to progressive drought stress by partially compensating for water loss from transpiration. Root tissues from the control as well as drought stress plants were harvested when the normalized transpiration ratio reached ∼0.1 in the stressed plants. After harvesting, soil particles were removed from the roots using 70% ethanol, and the root tissue was frozen in liquid nitrogen and stored at −80⁰C until RNA/protein/metabolite extraction.

### 2.2 RNA extraction and transcriptome sequencing

Total RNA from chickpea root tissues of four genotypes (ICC 4958, JG 11, JG 11+, and ICC 1882) harvested under control and drought stress conditions was isolated using “NucleoSpin^®^ RNA Plant” kit (Macherey‐Nagel) according to the manufacturer's instructions. The qualitative and quantitative assessment of the total RNA samples was conducted using an Agilent 2100 Bioanalyzer (Agilent Technologies) and a Nanodrop 8000 Spectrophotometer (Thermo Scientific).

Paired‐end sequencing was performed in‐house (Sequencing and Informatics Services Unit, Center of Excellence in Genomics & Systems Biology, ICRISAT) using Illumina HiSeq 2500 sequencing platform. The raw reads obtained from the sequencing of all eight samples were subjected to quality filtering using NGS‐QCbox (Katta et al., 2015) and Trimmomatic v0.35 (Bolger et al., 2014). The low‐quality reads (Phred score < 30; read length < 50 bases) and reads with adapter contamination were removed to generate a set of high‐quality reads/clean data. The downstream analysis was conducted based on clean data.

**Core Ideas**

- Multi‐omics analysis of chickpea roots revealed complex molecular mechanisms underpinning drought stress response.
- Integration of transcriptome and proteome data uncovered hub proteins involved in drought stress response pathways.
- Metabolomic profiling identified six metabolites showing a significant correlation with galactose metabolism.
- Transcriptome‐proteome integration revealed prominent differential expression of key genes underlying the *“QTL‐hotspot”* region.

The filtered high‐quality reads were mapped to the chickpea reference genome (Varshney, Song, et al., 2013) using Tophat v2.1.0 (Kim et al., 2013). The mapped reads for each sample were assembled using Cufflinks v2.2.1 (Trapnell et al., 2012) to generate reference‐guided assemblies, followed by merging of the assemblies to generate a consensus assembly using Cuffmerge. The consensus assembly was used for all downstream analyses. Transcript abundance was estimated based on fragments per kilobase of transcript per million mapped reads (FPKM). Transcripts with FPKM ≥ 1 in any of the samples and quantification status as “OK” were only considered to be expressed and were considered for further analysis. Global gene expression analysis and hierarchical clustering was performed using the “pheatmap” package in R software. Transcripts with FPKM ≥ 1 were log_2_ +1 transformed and hierarchical clustering was performed. Samples were further clustered based on their pairwise correlations. The differentially expressed genes (DEGs) were identified using Cuffdiff (Trapnell et al., 2010). The expression of the genes was estimated in terms of FPKM. A gene was considered to be differentially expressed if it exhibited a log_2_ fold change of ≥1.5 (upregulated) or ≤−1.5 (downregulated) and FPKM ≥ 3 (genes with high expression group) in each of the combinations studied. The putative functions of the identified DEGs were determined by subjecting the DEGs to blastx similarity searches (*E*‐value > 1e‐05) against NCBI nonredundant *Viridiplantae* protein database. Further, GO annotations were assigned to the genes using BLAST2GO (Conesa et al., 2005).

### 2.3 Protein extraction and proteomic analysis

Proteins were extracted and analyzed as described in Chaturvedi et al. (2015). Proteins were pre‐fractionated by SDS‐PAGE. Forty micrograms of total protein was loaded onto a gel. Gels were fixed and stained with methanol:acetic acid:water:Coomassie Brilliant Blue R‐250 (40:10:50:0.001). Gels were destained in methanol:water (40:60). Gel pieces were destained, equilibrated and digested with trypsin, desalted, and concentrated according to Valledore and Weckwerth (2014). Prior to mass spectrometric measurement, the tryptic peptide pellets were dissolved in 4% (v/v) acetonitrile and 0.1% (v/v) formic acid. One microgram of each sample was loaded on a C18 reverse phase column (Thermo Scientific, EASY‐Spray 500 mm, 2 µm particle size). Separation was achieved with a 90 min gradient from 98% solution A (0.1% formic acid) and 2% solution B (90% ACN and 0.1% formic acid) at 0 min to 40% solution B (90% ACN and 0.1% formic acid) at 90 min with a flow rate of 300 nL min^−1^. nESI‐MS/MS measurements were performed on Orbitrap Elite (Thermo Fisher Scientific) with the following settings: Full scan range 350–1800 m/z resolution 120000, max. 20 MS2 scans (activation type collision‐induced dissociation), repeat count 1, repeat duration 30 s, exclusion list size 500, exclusion duration 30 s, charge state screening enabled with the rejection of unassigned and +1 charge states, and minimum signal threshold 500.

Raw data were searched with the SEQUEST algorithm present in Proteome Discoverer version 1.3 (Thermo Fisher Scientific) as described in Chaturvedi et al. 2013. We have used the following settings in Proteome Discoverer for data analysis which include peptide confidence: high, which is equivalent to 1% false discovery rate (FDR), and Xcorr of 2, 3, 4, 5, 6 for peptides of charge 2, 3, 4, 5, 6. For identification, annotated chickpea genome database containing 27,494 protein annotations (Varshney, Song, et al., 2013) was used. The identified proteins were quantitated based on total ion count, followed by a normalized spectral abundance factor (NSAF) normalization strategy (Paoletti et al., 2006).

Hierarchical clustering of Pearson's correlation was used to study pairwise correlations in all eight samples studied. The replicated normalized protein data were used for the identification of differentially expressed proteins (DEPs). The *t*‐test was used to check the significant difference in mean for individual proteins in each genotype under controlled and stress conditions. The proteins showing significantly different expression (*p* ≤ 0.05) under controlled and stress condition were considered as DEPs and used for downstream analysis. Venn diagrams were generated using GennVenn (http://genevenn.sourceforge.net/) to visualize proteome regulation between control and stress conditions in different genotypes.

### 2.4 Metabolite profiling

Metabolites extraction was performed with slight modifications according to the protocol and all analysis steps, including sample derivatization and gas chromatography coupled to time‐of‐flight mass spectrometry (GC‐TOF‐MS) were carried out as previously described (Weckwerth et al., 2004).

Data analysis was performed using ChromaTof (Leco) software. Chromatograms of different samples were used to generate a reference peak list, and all other data files were processed against this reference list. Retention index markers were used to calculate retention indices of compounds and for chromatographic alignment. Deconvoluted mass spectra were matched against an in‐house mass spectral library, and this retention index was also used for peak annotation. Peak annotations, as well as peak integrations, were checked manually before exporting peak areas for relative quantification into a Microsoft Excel program. Areas of different trimethylsilyl (TMS) derivatives of single metabolites were summed, and from methoxyamine products, only one peak was selected for further analyses. All hydrophilic metabolite amounts are given in arbitrary units corresponding to the peak areas of the chromatograms (Wang, Fu, et al., 2016).

### 2.5 Signaling pathway analysis and multi‐omics data integration

The expressed DEGs (log_2_ fold change ≥ 1.5 or ≤ −1.5) correlating with expressed DEPs (fold change ≥ 1.5 or ≤ −1.5) were mapped to their respective pathways using Kyoto Encyclopedia of Genes and Genomes (KEGG) Automatic Annotation Server (Moriya et al., 2007). Multi‐omics data integration was carried out keeping proteome data in the center. Initially, the DEPs from all the genotypes were combined to generate a nonredundant set of DEPs. Later, expression values of each protein (fold change ≥1.5 or ≤−1.5) and gene (log_2_ fold change ≥1.5 or ≤−1.5) under control and stress conditions were calculated and used for integrative analysis. In order to integrate transcriptome and metabolome data, activated signaling pathways were correlated with the primary metabolites identified.

### 2.6 Quantification and statistical analysis

The heat maps were generated based on the hierarchical clustering of Pearson's correlations (*R*). Technical and biological replicate experiments were performed as indicated.

## 3 RESULTS

To unravel the changes in transcript, protein, and metabolite levels in response to drought stress in chickpea roots, an integrative multi‐omics approach was followed. This study targeted the identification of key genes and molecular mechanisms underlying drought stress response in chickpea. Here, four chickpea genotypes contrasting for drought stress response (ICC 4958, JG 11, and JG 11+ [drought‐tolerant], and ICC 1882 [drought‐sensitive]) were compared to find out DEGs, DEPs, and differentially occurring metabolites. An integrated analysis of transcriptome, proteome, and metabolome data provided a deeper understanding of the signaling pathways and candidate genes involved in drought stress response mechanisms in chickpea.

### 3.1 Transcriptome profiling

A total of 364.5 million (M) raw reads were generated from the paired‐end sequencing of four chickpea genotypes under control and stress conditions. After stringent quality filtering, 341.1 M (94%) of the 364.5 M raw reads representing high‐quality reads were processed for mapping onto the chickpea reference genome (Varshney, Song, et al., 2013). On average, about 89% (304.6 M) of the high‐quality reads were mapped to the chickpea genome. The sample wise details of sequence data generated, filtered reads, reads mapped on the genome, and percent alignment is given in Table S1. For ease of understanding, the samples hereafter have been designated as genotype_condition. For example, the sample ICC 4958_C denotes genotype ICC 4958 under control conditions, and ICC 4958_S denotes genotype ICC 4958 under drought stress conditions.

**FIGURE 1:**
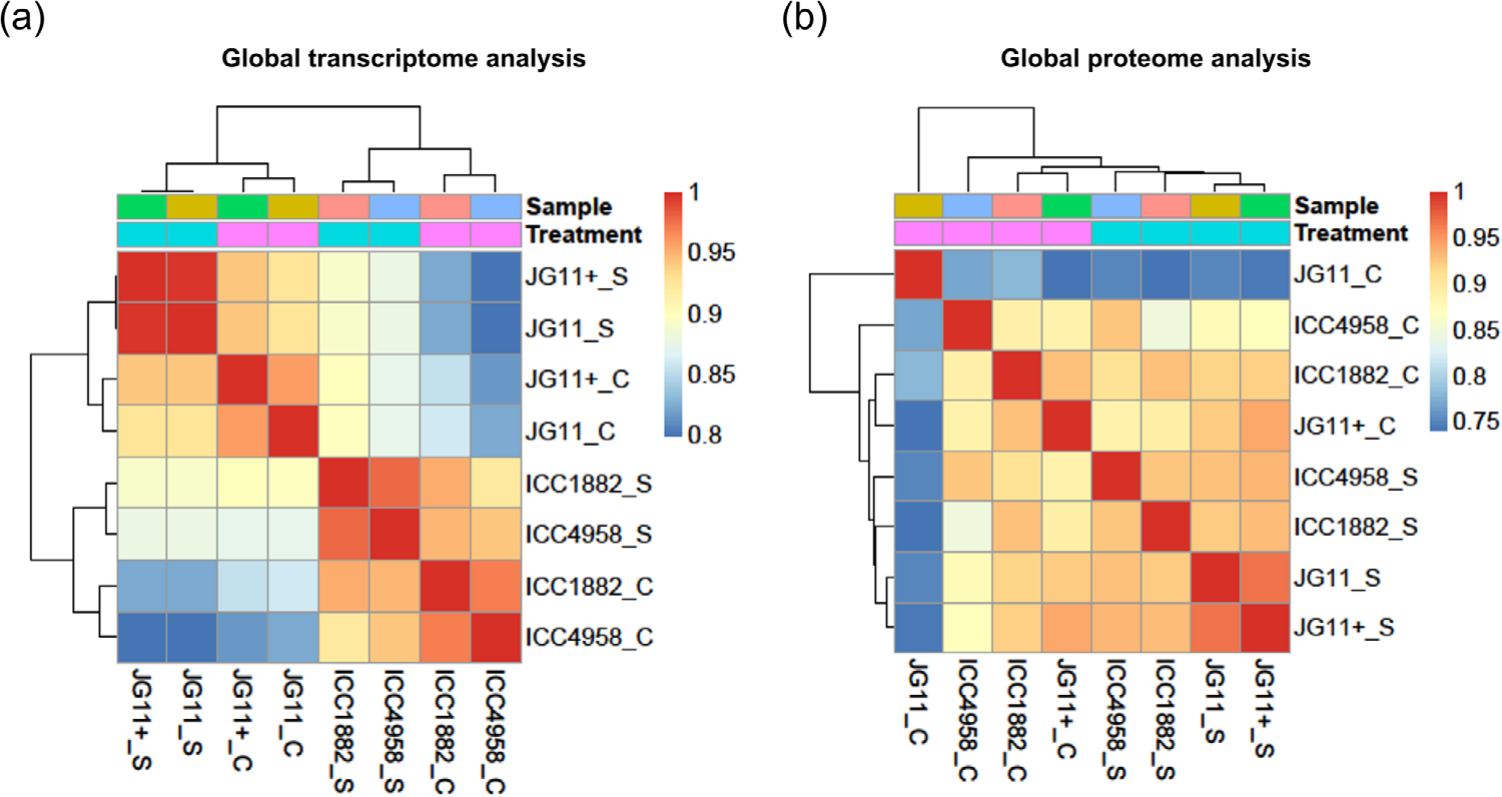
Global transcriptome and proteome analysis among root tissues of contrasting drought stress‐responsive chickpea genotypes. Heatmaps for (a) transcriptome and (b) proteome profiling have been shown based on hierarchical clustering of Pearson's correlations (*R*) for the eight samples. The color scale indicates the degree of correlation. Samples were clustered based on their pairwise correlations. In the global transcriptome analysis (a), genes with normalized expression level FPKM > 1 in at least one of the eight samples analyzed were designated as expressed and shown in the figure. In the global proteome analysis (b), proteins that are present in at least one of the genotypes under control or stress conditions were considered as expressed and are shown here.

The mapped reads (304.6 M) on the chickpea genome were used to generate reference‐guided assembly, and to analyze global and differential gene expression profiles. The reference‐guided assembly generated 32,217 transcripts and its comparison with the chickpea genome led to the identification of 27,894 (87%) annotated and 4,323 (13%) novel transcripts. The normalized expression level, FPKM of each transcript, was estimated in all eight samples. To exclude transcripts with low confidence expression values, only those transcripts with an FPKM ≥ 1 in at least one of the samples analyzed were designated as expressed. Based on these criteria, a total of 22,707 (70%) out of 32,217 transcripts were identified to be transcriptionally active in the present dataset (Table S2), hereafter referred to as genes. Transcriptional activity was variable and diverse across all the samples studied, with ICC 1882_S expressing the largest number (19,458; 86%) and JG 11_S expressing the smallest number (18,070; 80%) of genes. Based on the expression levels, the genes were categorized as low/moderately expressed (FPKM ≥ 1 and ≤ 10) and highly expressed (FPKM > 10). Among all the samples, ICC 1882_C followed by ICC 1882_S and ICC 4958_S showed a maximum number of highly expressed genes (FPKM > 10). Low/moderately expressed genes (FPKM ≥ 1 and ≤ 10) were maximum in ICC 4958_S, followed by JG 11_S and ICC 1882_S. The distribution of the expressed genes across the eight samples under these categories has been provided in Table S3. A subset of 15,473 (48%) transcripts out of 22,707 were identified to be transcriptionally active (FPKM ≥1) in all the samples studied. Further, hierarchical clustering of Pearson's correlation in the transcriptome data well reflected the biological identity of the eight samples (Figure 1a). The tissues clustered together were genotype and condition (control/stress) specific. For example, samples of ICC 4958 and ICC 1882 genotypes were clustered together, whereas JG 11 and JG 11+ genotypes formed different cluster. Control samples of both the genotypes in each of the main clusters were sub‐clustered and stress samples formed another sub‐cluster. Further, gene ontology (GO) analysis was performed on the transcriptionally active genes in all the samples, which indicated an over‐representation of the following GO terms: ATP binding (1897), transport (1523), deoxyribonucleic acid (DNA) binding (1321), RNA binding (1199), electron transport (158), defense response (87), and so on. These results indicate a substantial and comprehensive representation of the transcriptome.

The DEGs were identified in each of the four genotypes by comparing drought stress sample to the respective control sample. The DEG data analysis identified a total of 3956 unique set of DEGs across four genotypes (Table S4). Data analysis of each genotype, that is ICC 4958, JG 11, JG 11+, and ICC 1882, identified 1704, 1835, 1245, and 1308 DEGs, respectively (log_2_ fold change ≥1.5 or ≤1.5). Further, induced and repressed genes in each genotype—ICC 4958 (598 and 1106), JG 11 (590 and 1245), JG 11+ (488 and 757), and ICC 1882 (511 and 797)—were identified. There were 2136 DEGs common for two or more genotypes, while 717, 862, 358, and 401 DEGs were unique to ICC 4958, JG 11, JG 11+, and ICC 1882, respectively. Furthermore, DEGs during drought stress conditions in the four chickpea genotypes were evaluated (Figure S2). In total, 173 DEGs were found to be differentially expressed between ICC 4958 and JG 11+. Similarly, 764 genes were differentially expressed between JG 11 and JG 11+, while 289 DEGs were common between ICC 4958, JG 11, and JG 11+ (Table S5).

### 3.2 Proteome profiling

Comparative proteome analysis was performed to identify root tissue‐specific protein signatures in all four genotypes of chickpea (ICC 4958, JG 11, JG 11+, and ICC 1882) under control and drought stress conditions. A total of 4252 proteins were identified, and further analyses were performed by considering the proteins that are present in at least one of the genotypes under control or stress conditions, which lead to the subset of 2751 proteins (Table S6). Hierarchical clustering of Pearson's correlation of these 2751 proteins revealed treatment‐specific biological identity (Figure 1b). The tissues clustered together were treatment (control/stress) specific and irrespective of the genotype. For instance, samples of all four genotypes under control conditions were clustered together, whereas the remaining four samples from stress conditions formed a different cluster. Among the stress samples, JG 11 and JG 11+ formed sub‐cluster.

In order to generate a broad survey of identified proteins with altered genotype‐specific regulations under drought stress in chickpea, a Venn analysis was conducted that determines the dynamics of the proteome (Figure S3a). The genotype‐specific DEPs identified are represented using volcano plots (Figure S3b). Based on the expression levels, the DEPs displayed a significant change in abundance (*p* < 0.05) in the four genotypes. Regulation of the proteome was enhanced under stress conditions compared to control in all the genotypes. In ICC 4958, proteins such as annexin (*Ca_26048* and *Ca_20270*) and vacuolar H+ ATPase V0 (*Ca_20692*) were identified and showed increased levels of expression under stress conditions compared to its control (Table S6). Further, proteins showing higher expression under stress conditions, for example, dehydrin (*Ca_12999*), ABA‐induced protein (*Ca_08450*), and so on were also identified in ICC 4958. Similarly, JG 11 also showed enhanced regulation of protein candidates such as glutathione S‐transferase (*Ca_13294*), calcium‐transporting ATPase 8 (*Ca_22107*) under stress conditions. On the other hand, JG 11+ showed upregulation of aspartate aminotransferase (*Ca_13475*), auxin binding protein (*Ca_10011*), acidic endochitinase like protein (*Ca_04407* and *Ca_ 11655*), under stress compared to control condition. Interestingly, a distinct class of proteins such as alpha‐mannosidase (*Ca_10568*), L‐ascorbate peroxidase (*Ca_11025*), and ethylene‐insensitive protein (*Ca_12043*), showed enhanced regulation in the sensitive genotype ICC 1882 under drought stress compared to control (Table S6).

Unique DEPs in each genotype were examined which led to the identification of 160, 331, 127, and 120 DEPs in ICC 4958, JG 11, JG 11+, and ICC 1882, respectively. For example, serine/threonine‐protein phosphatase (*Ca_05784* and *Ca_18208*), flowering‐promoting factor 1‐like protein 2 (*Ca_10972*), hsp70 nucleotide exchange factor fes1‐like (*Ca_21949*), late embryogenesis abundant (LEA) 4 protein (*Ca_07309*), and so on were identified only in ICC 4958, whereas cysteine rice receptor kinase (*Ca_20192* and *Ca_11895*), DEAD‐box ATP‐dependent RNA helicase‐like protein (*Ca_02750* and *Ca_22290*), leucine‐rich repeat (LRR) receptor‐like kinase (*Ca_13768* and *Ca_06625*), root hair defective 3 GTP‐binding family protein (*Ca_10083*), and so on were identified in JG 11. In JG 11+, glyoxysomal fatty acid beta‐oxidation MFP‐A protein (*Ca_14746* and *Ca_02963*), ras‐related protein (*Ca_20398* and *Ca_17202*), among others were identified. The sensitive genotype ICC 1882 showed differential regulation of glucan endo‐1,3‐beta‐glucosidase (*Ca_27887* and *Ca_00644*), 60S ribosomal protein (*Ca_02590*, *Ca_07452*, and *Ca_17790*), aspartate aminotransferase (*Ca_09590* and *Ca19888*) and so on These genotype‐specific DEPs demonstrate potential proteins which are involved in drought stress response mechanism and aspects of complex plant physiological and biochemical variations, especially during stress conditions.

**FIGURE 2:**
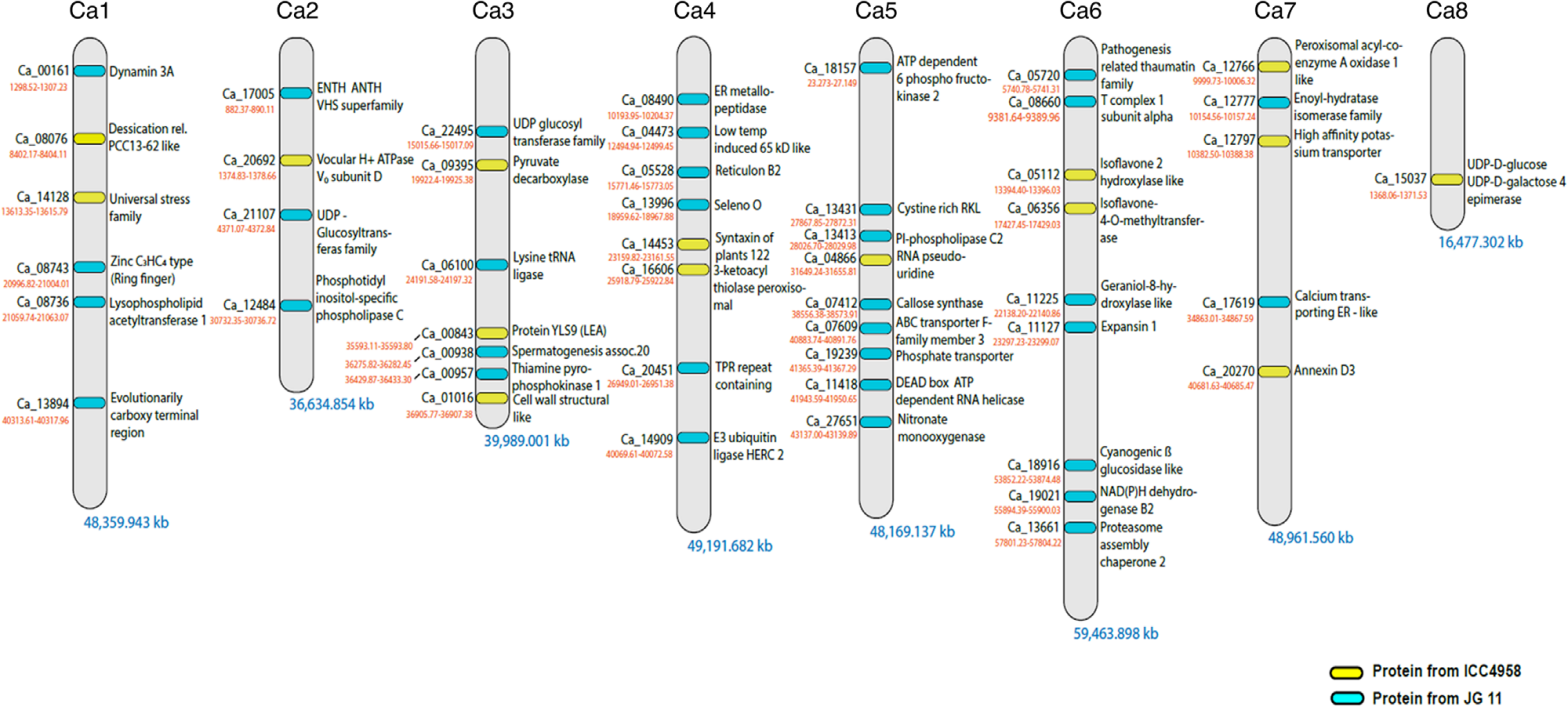
Chromosomal distribution of drought stress responsive proteins in the introgression line JG 11+. The figure shows chromosomal distribution of all differentially expressed proteins (DEPs) between control and drought stressed root tissues of JG 11+ genotypes. Of these DEPs, proteins encoded from the introgressed genomic segments from ICC 4958 (in the genetic background of JG 11) have been shown in yellow, while the proteins encoded from genomic segments from JG 11 have been shown in blue. The chromosome number is indicated at the top of each chromosome.

To understand and predict the inheritance of drought stress response mechanisms in JG 11+, a comparative analysis of DEPs among ICC 4958, JG 11, and JG 11+ was conducted. Inherited DEPs from ICC 4958 and JG 11 into JG 11+ were identified and mapped onto different chickpea chromosomes (Figure 2). The identified DEPs from ICC 4958 and JG 11 span across all eight chickpea chromosomes. In total, 15 DEPs were common between ICC 4958 and JG 11+ (Figure S4a). The majority of the DEPs that were predicted to be inherited from ICC 4958 formed an integral component of the membrane and are hypothesized to play a role in protecting its stability during dehydration. These include LEA (predicted YLS9 protein) (*Ca_00843*), isoflavone 2‐hydroxylase‐like (*Ca_05112*), desiccation‐related PCC13‐62‐like (*Ca_08076*), high‐affinity potassium transporter (*Ca_12797*), and syntaxin of plants 122 (*Ca_14453*). Furthermore, increase in the regulation levels of the proteins, LEA (1.6‐fold in ICC 4958 and 2.1‐fold in JG 11+) and LEA‐like desiccation‐related protein PCC13‐62 (4.4‐fold in ICC 4958 and 2.5‐fold in JG 11+), was observed under stress condition. The highly upregulated proteins in ICC 4958 and JG 11+ under stress conditions included UDP‐d‐glucose UDP‐d‐galactose 4‐epimerase (*Ca_15037*) (53‐fold and 37‐fold, respectively) (Figure S4a). This set of DEPs indicates the inheritance of drought tolerance from ICC 4958 into JG 11+.

A total of 36 DEPs were common between JG 11 and JG 11+ (Figure S4b). Highly upregulated proteins included low‐temperature‐induced 65 kDa‐like isoform (*Ca_04473*), pathogenesis‐related thaumatin family protein (*Ca_05720*), UDP‐glucosyltransferase family protein (*Ca_21107* and *Ca_22495*), cyanogenic beta‐glucosidase‐like (*Ca_18916*), expansin A1 (*Ca_11127*), thiamine pyrophosphokinase 1 (*Ca_00957*), proteasome assembly chaperone 2 (*Ca_13661*), and geraniol 8‐hydroxylase‐like (*Ca_11225*), among others. Plant cell membranes serve as the primary site of perception of external abiotic stimuli, thereby activating a cascade of downstream signaling. Several integral membrane proteins including reticulon B2 (*Ca_05528*), endoplasmic reticulum metallopeptidase (*Ca_08490*), lysophospholipid acyltransferase 1 (*Ca_08736*), zinc C3HC4 type (RING finger) (*Ca_08743*), cysteine‐rich RLK (*Ca_13431*), calcium‐transporting endoplasmic reticulum‐type (*Ca_17619*), and high‐affinity inorganic phosphate transporter (*Ca_19239*) were differentially expressed under drought stress. Further, a cell wall biosynthesis and metabolism protein, callose synthase (*Ca_07412*), and two proteins from the UDP‐glucosyltransferase family (*Ca_21107* and *Ca_22495*) were also upregulated in JG 11 and JG 11+ (Figure S4b).

In total, nine DEPs were common among all three tolerant genotypes (ICC 4948, JG 11, and JG 11+) (Figure S4c). These included CoA ligase‐like protein (*Ca_02566*), acyl‐CoA dehydrogenase IBR3 (*Ca_14755*), l‐gulonolactose oxidase (*Ca_01350*), delta‐1‐pyrroline‐5‐carboxylate synthesis (*Ca_24241*), binding protein (*Ca_20001*), phospholipase A1 (*Ca_00378*), and dehydrin DHN3‐like (*Ca_12999*), which indicated the presence of some common drought regulated genes.

### 3.3 Transcriptome‐proteome correlation analysis

**FIGURE 3:**
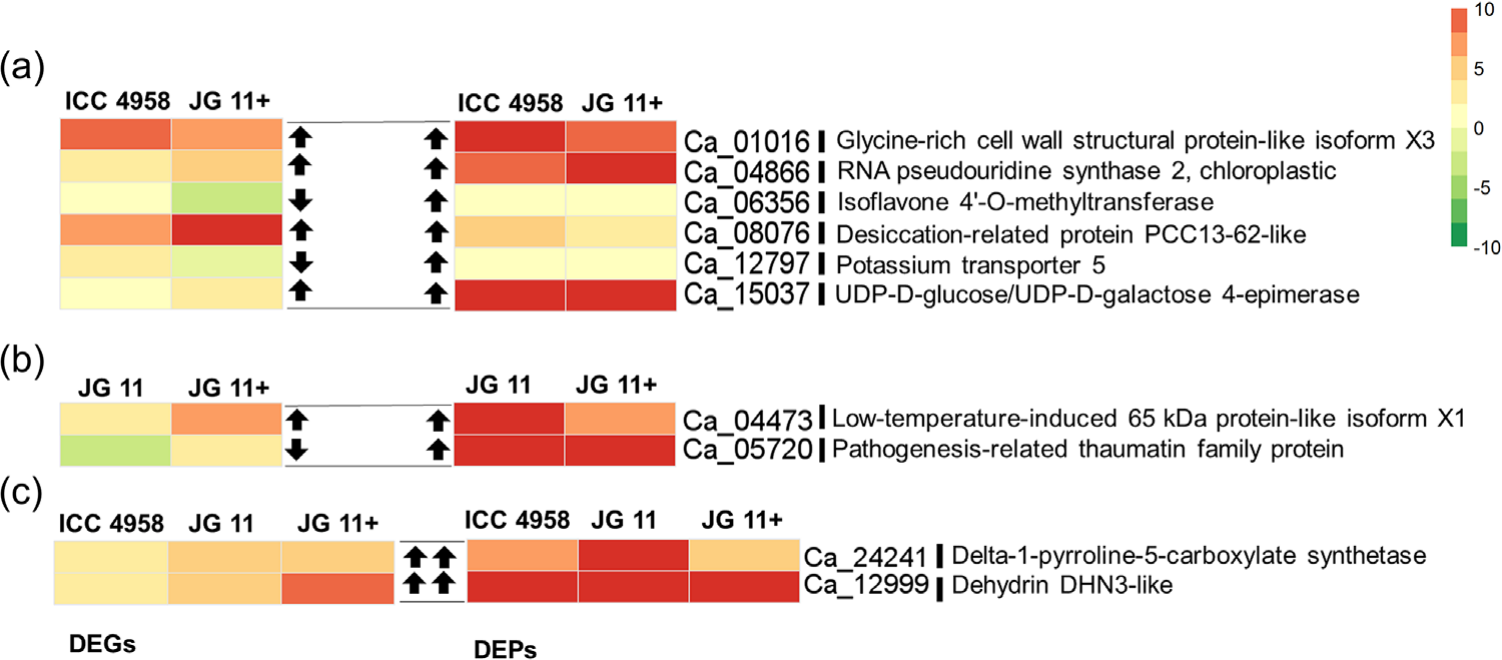
Expression profiles of correlated differentially expressed genes (DEGs) and differentially expressed proteins (DEPs). Combined view of the expression levels of DEGs and their correlated DEPs in the two tolerant genotypes and the introgression line. The genotype combinations were (a) ICC 4958 and JG 11+, (b) JG 11 and JG 11+, and (c) ICC 4958, JG 11, and JG 11+. The heat map on the left indicates the DEGs expression and the heat map on the right indicates the corresponding DEPs expression. The arrows (**↑** and **↓**) indicates up‐ and downregulation of the corresponding DEGs and DEPs in the genotype combinations

The expression of genes and their target proteins were integrated to infer the role of key proteins involved in drought stress response mechanisms. One‐to‐one correlation analysis led to the identification of 900 proteins out of 2751 proteins, which had their corresponding genes in the transcriptome data of all four genotypes with ≤−1.5 or ≥1.5 log_2_ fold change. The DEGs above a threshold level of expression, which have the potential to influence the phenotype or functioning of the cell, were considered for analysis. Among the 15 proteins common for ICC 4958 and JG 11+, six proteins showed corresponding DEGs when one‐to‐one correlation was performed (Figure 3a). The six proteins included glycine‐rich cell wall structural‐like isoform (*Ca_01016*), RNA pseudourine (*Ca_04866*), isoflavone 4‐O‐methyltransferase (*Ca_06356*), desiccation‐related PCC13‐62‐like (*Ca_08076*), high‐affinity potassium transporter (*Ca_12797*), and UPD‐d‐glucose/UDP‐d‐galactose 4‐epimerase (*Ca_15037*). These represented a suite of genes involved in cell wall organization, transmembrane transport, protein dimerization, RNA binding, and cell metabolism. Major protein‐coding genes involved in cell wall modification include structural proteins, membrane‐binding proteins, and transmembrane transporters. Interestingly, glycine‐rich cell wall structural‐like, desiccation‐related PCC13‐62‐like, and UDP‐d‐glucose/UDP‐d‐galactose 4‐epimerase proteins were highly induced, whereas high‐affinity potassium transporter protein was repressed in ICC 4958 and JG 11+. Significant upregulation was observed in desiccation‐related PCC13‐62‐like protein.

Among the 36 DEPs common for JG 11 and JG 11+ genotypes, a one‐to‐one correlation identified two DEPs that possessed their corresponding DEGs in the transcriptome data. These included low temperature induced 65 kDa‐like isoform (*Ca_04473*) and pathogenesis‐related thaumatin family protein (*Ca_05720*) (Figure 3b). Furthermore, among the nine DEPs common for all three tolerant genotypes ICC 4958, JG 11, and JG 11+, a one‐to‐one correlation identified two DEPs, delta‐1‐pyrroline‐5‐carboxylate synthetase, and dehydrin DHN3‐like, corresponding to the DEGs in the transcriptome data (Figure 3c). Delta‐1‐pyrroline‐5‐carboxylate synthetase, which is involved in proline biosynthesis, and dehydrin DHN3‐like protein, which represents a group of LEA II proteins, were found to be differentially expressed. Interestingly, delta‐1‐pyrroline‐5‐carboxylate synthetase (*Ca_24241*) was found to be differentially expressed in all three genotypes except the drought‐sensitive genotype (ICC 1882), indicating its major role in the drought tolerance mechanism.

### 3.4 Regulation of drought stress‐responsive signaling pathways

Pathway enrichment analysis was performed to investigate the major pathways significantly altered/activated under drought stress. The expressed DEGs showing one‐to‐one correlation with regulated DEPs (900) were subjected to pathway analysis. A total of 75 significantly enriched pathways representing 175 enzymes were found to be activated. The number of enzymes identified in each pathway ranged from 1 to 15, and a total of 111 unique genes were involved in one or more of the pathways identified. Major pathways activated include biosynthesis of antibiotics (18 genes), phenylpropanoid biosynthesis (13), starch and sucrose metabolism (12), pyruvate metabolism (10), galactose metabolism (7), inositol phosphate metabolism (6), glycolysis/ gluconeogenesis (6), glycerolipid metabolism (6), amino sugar and nucleotide sugar metabolism (6), cysteine and methionine metabolism (5), glycerophospholipid metabolism (5), pentose and glucoronate interconversions (5), isoflavonoid biosynthesis (4), methane metabolism (4), isoquinoline alkaloid biosynthesis (4), and glycine, serine and threonine metabolism (4).

In the present study, biosynthesis of antibiotics pathway was identified as one of the major pathways involving kinase (EC 2.7.2.11), dehydrogenases (EC 1.1.1.37, EC 1.1.1.95, EC 1.2.1.3, EC 1.1.1.2, EC 1.2.1.41), carboxykinases (EC 4.1.1.32, EC 4.1.1.49), phosphatase (EC 3.1.3.3), diphosphate synthase (EC 2.5.1.10), and decarboxylase (EC 4.1.1.33), among others. Kinases and dehydrogenases play a central role in signaling during plant defense mechanisms. Further, plant secondary metabolic gene clusters implicate their role in the synthesis of defense compounds, which include genes for the synthesis of anthocyanin, antibiotics, defense compounds, and so on. Similarly, phenylpropanoid biosynthetic pathway involving gentiobiase (EC 3.2.1.21), reductase (EC 1.2.1.44), lactoperoxidase (EC1.11.1.7), and O‐methyltransferase (EC 2.1.1.104) is of central importance and is well known to be activated under abiotic stress response (such as drought, salinity, and high/low temperature) resulting in accumulation of various phenolic compounds, which have a potential to scavenge harmful reactive oxygen species (ROS).

This study also revealed regulation of galactose metabolism pathway involving the enzymes, epimerase (EC 5.1.3.2), galactosyltransferase (EC 2.4.1.82), 3‐alpha‐galactosyltransferase (EC 2.4.1.123), lactase (EC 3.2.1.23), and reductase (EC 1.1.1.21). The importance of epimerase and galactosyltransferase enzymes in cell wall biosynthesis and the growth of cell walls has been clearly recognized. Galactosyltransferases are also known to regulate stress‐related gene expression by maintaining endomembrane organization and minimizing cellular damage limiting the ROS concentration in the cell.

Similarly, regulation of the isoflavonoid biosynthesis pathway was identified in the present study and involved methyltransferases (EC 2.1.1.212, EC 2.1.1.270, and EC 2.1.1.150) and malonyltransferase (EC 2.3.1.115). Flavonoids are known for their significant contribution to plant defense against environmental stresses. In the process, methyltransferases enhance the biosynthesis of 4′O‐methylated isoflavonoid phytoalexins and are known to differentially modify isoflavone glucosides in plants under various stresses. The involvement of these pathways strongly indicate changes in gene/protein expression profiles during drought stress in chickpea. Details of pathways are identified and their corresponding genes identified are given in Table S7 and Figure S5.

### 3.5 Metabolome profiling

**FIGURE 4:**
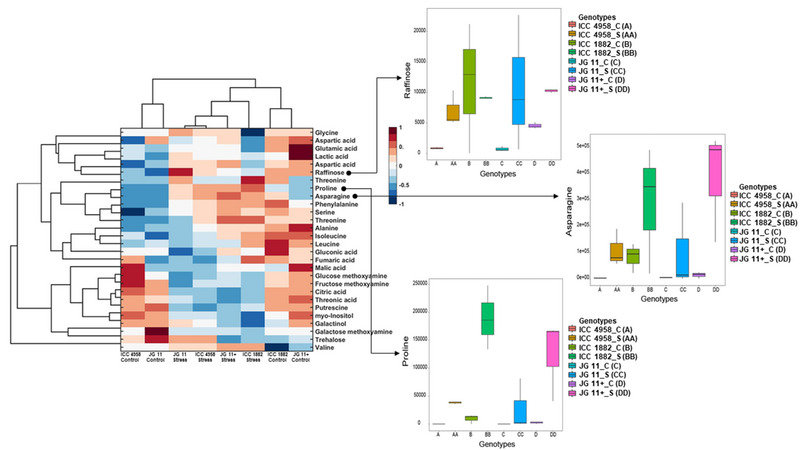
Bi‐clustering heat map analysis of the identified metabolites in chickpea genotypes. Boxplots provides genotypic regulation of the raffinose, proline, and asparagine in the root tissues under control and stress condition. Graphical representation of boxplots indicates a considerable increase in the accumulation of amino acids in the root tissues under drought stress when compared to control conditions.

In order to understand accumulation and changes in the metabolites under drought stress, metabolomic profiling was carried out under control and drought stress‐challenged root tissues of the four chickpea genotypes. A total of 34 metabolites were identified and were highly reproducible in four different genotypes. The identified metabolites included amino acids, organic acids, and sugars. A multivariate statistical analysis of the metabolite data matrix (Table S8) allowed an unbiased view of root metabolism, and therefore, a principal component (PC) analysis was performed (Figure S6). Control and stress conditions were separated on the first PC (PC1), which explained 34.97% of the total variation, whereas the second component (PC2) explained 22.22% variation across the data set. Considering the loadings between PC1 and PC2, two distinct groups associated with control and stress samples were identified, which suggested a clear distinction in the metabolite accumulation under the two conditions. Further, the effect of drought stress conditions on metabolic accumulation becomes evident from hierarchical clustering with the heat map (Figure 4). For generating a bi‐cluster, all the TMS derivatives were summed up. Drought stress imposition considerably increased the accumulation of amino acids such as proline, valine, threonine, serine, and asparagine in all the cultivars. Interestingly, raffinose showed increased levels only in the tolerant genotypes (ICC 4958, JG 11, and JG 11+) compared to the sensitive genotype ICC 1882 (Figure 4). A total of 26 metabolites, viz., alanine, asparagine, aspartic acid, citric acid, fructose, galactinol, gluconic acid, glycine, and isoleucine were mapped to different pathways analyzed, which include citrate cycle metabolism, galactose metabolism, glycine, serine, and threonine metabolism (Table S7).

### 3.6 Integrated transcriptome‐proteome‐metabolome analysis

**FIGURE 5:**
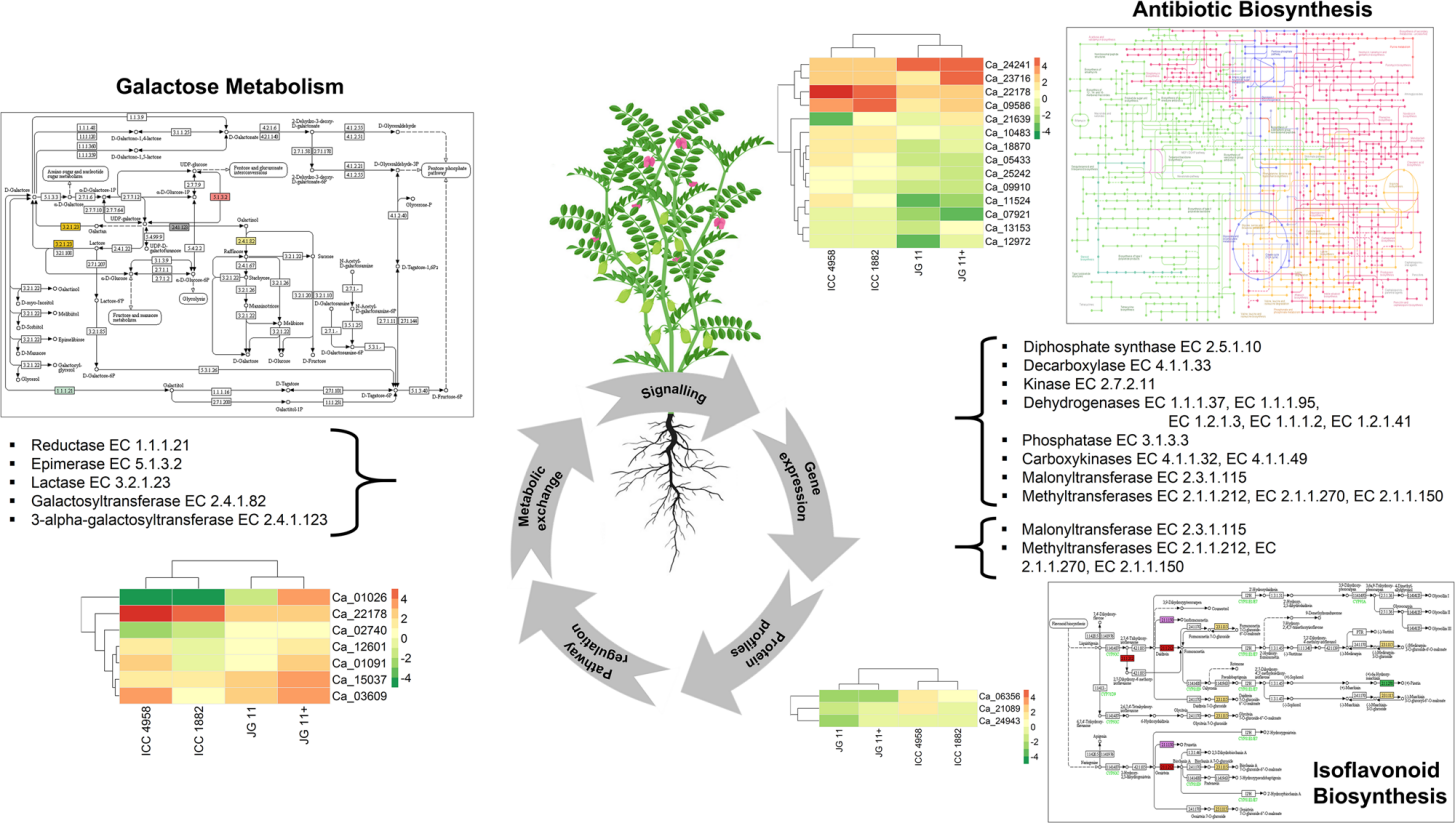
Major signaling pathways altered in chickpea roots and associated with drought stress response mechanism. Key genes identified through integrated‐omics data analysis and expression patterns of corresponding enzymes in the four chickpea genotypes were shown as heatmaps against respective enriched pathways.

Expression patterns of genes, proteins, and the regulated pathways together with associated metabolites under drought conditions were investigated through an integrative multi‐omics approach. The integrated transcriptomics and proteomics data revealed significant differences in gene and protein expression patterns between contrasting drought stress‐responsive chickpea genotypes, ICC 4958, JG 11, JG 11+ (tolerant), and ICC 1882 (sensitive) under drought stress. In summary, a total of 15 and 36 proteins from the tolerant genotypes ICC 4958 and JG 11, respectively, were identified to be differentially regulated and were transferred into the IL JG 11+. On the other hand, nine differentially regulated proteins were found common between all three tolerant genotypes, which did not differentially express in the sensitive genotype ICC 1882. Of these 60 proteins which were differentially regulated in the tolerant genotypes under drought stress, a total of 10 proteins showed a correlation with corresponding DEGs from the transcriptome data. Here, six proteins were correlated between ICC 4958 and JG 11+, and two proteins each were identified between the other two combinations studied ([JG 11 and JG 11+] and [ICC 4958, JG 11, and JG 11+]). Of the 10 proteins which showed correlated expression both in transcriptome and proteome datasets, three proteins encoding isoflavone 4′‐O‐methyltransferase (*Ca_06356*), UDP‐d‐glucose/UDP‐d‐galactose 4‐epimerase (*Ca_15037*), and delta‐1‐pyrroline‐5‐carboxylate synthesis (*Ca_24241*) highlighted activation of targeted pathways under drought stress. The pathways of antibiotic biosynthesis, galactose metabolism, and isoflavonoid biosynthesis were mainly enriched and associated with drought tolerance mechanisms in different genotypes (Figure 5). Furthermore, six of the identified metabolites (fructose, galactose, glucose, myoinositol, galactinol, and raffinose) showed a significant correlation with galactose metabolism.

### 3.7 Combining multi‐omics data with the drought‐responsive *“* *QTL‐hotspot”* region

The identification of the *“* *QTL‐hotspot”* region on CaLG04 is considered a significant leap toward identifying the candidate genes associated with drought tolerance mechanism in chickpea (Varshney et al., 2014). Kale et al. (2015) refined the QTL‐hotspot region from ca. 3 Mb to two sub‐regions of 139.22 kb (*“QTL‐hotspot_a”*) and 153.36 kb (*“QTL‐hotspot_b”*), containing 26 annotated genes using bin mapping‐based QTL analysis and gene enrichment analysis. In order to clarify the role of *“* *QTL‐hotspot”* in imparting drought tolerance to JG 11+, expression patterns of the reported 26 genes were analyzed in the current transcriptome data. A total of 21 genes (14 genes from “*QTL‐hotspot*_*a*” and seven genes from “*QTL‐hotspot*_*b”* ) out of 26 genes were found to be expressed (FPKM ≥1) in the transcriptome data. Of these, three genes encoding aldo/keto reductase family oxidoreductase (*Ca_04551*), early light‐induced‐like protein (*Ca_04552*), protein TIFY 4A‐like isoform X6 (*Ca_04558*) from the “*QTL‐hotspot*_*a*” sub‐region; and three genes including LRR/extensin 2 (*Ca_04564*), kinase interacting (KIP1‐like) family protein (*Ca_04566*), and homocysteine S‐methyltransferase‐like protein (*Ca_04568*) from the “*QTL‐hotspot*_*b*” sub‐region showed differential regulation among the four genotypes studied.

**FIGURE 6:**
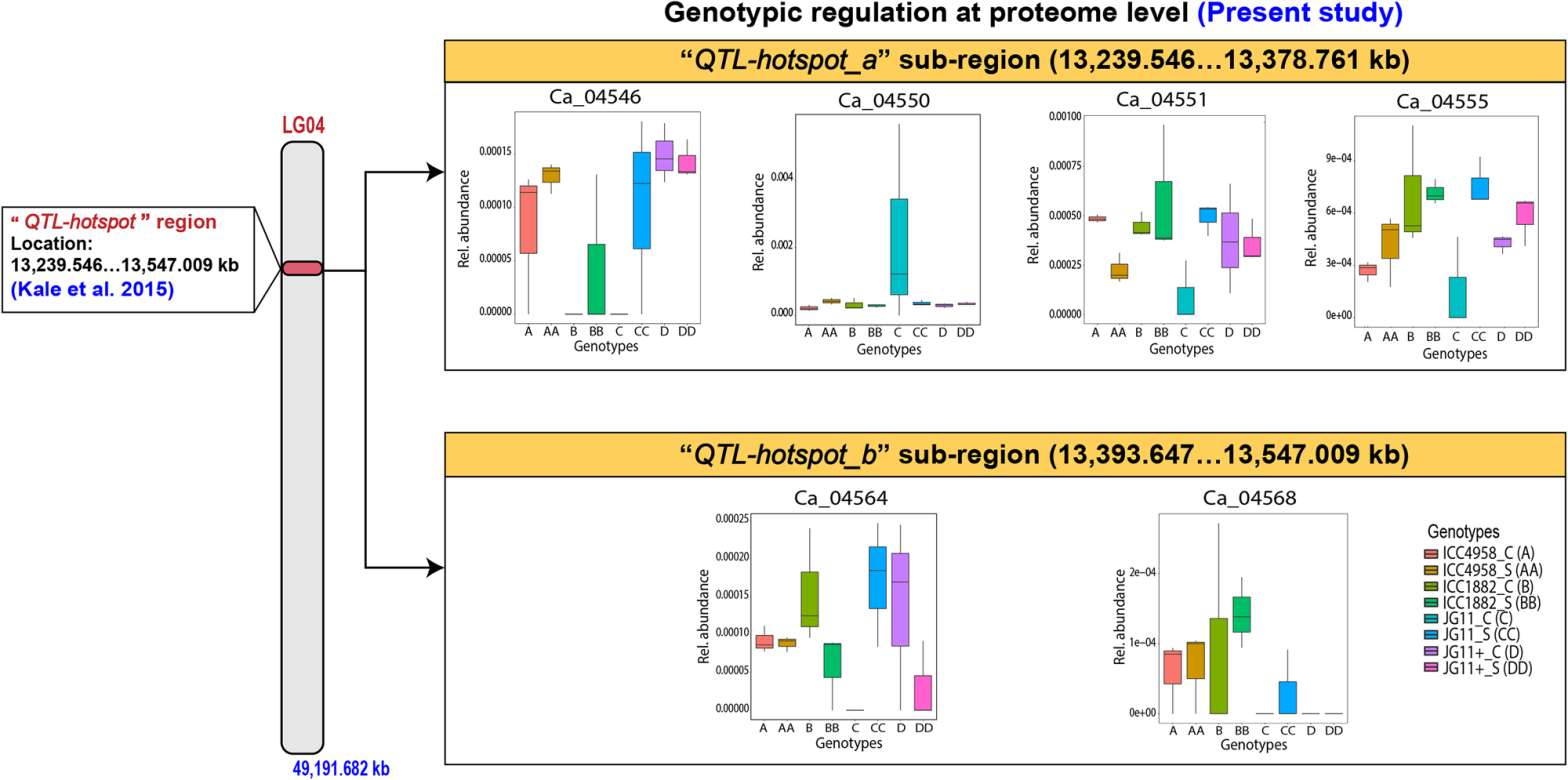
Genotypic regulation at the proteome level for the *“QTL‐hotspot”* genes under control and stress conditions. Boxplots represent the regulation of protein expression of four genes from “*QTL‐hotspot_a*” (above) and two genes from “*QTL‐hotspot_b*” (below). X‐axis represents the genotype and Y‐axis represents the relative abundance of proteins.

Furthermore, the expression patterns of proteins from the *“* *QTL‐hotspot”* region among all four genotypes were also compared. The expression of four genes including stem‐specific TSJT1 (*Ca_04546*), emp24 gp25L p24 family (*Ca_04550*), aldo keto reductase family oxidoreductase (*Ca_04551*), and 1,2‐dihydroxy‐3‐keto‐5‐methylthiopentene dioxygenase (*Ca_04555*) from *“QTL‐hotspot_a”*; and two genes including LRR extensin 2 (*Ca_04564*) and homocysteine S‐methyltransferase (*Ca_04568*) from *“QTL‐hotspot_b”* were found to be modulated at the proteome level (Figure 6). Among these 6 proteins, two (*Ca_04546* and *Ca_04555*) were upregulated, while one (*Ca_04551*) was down‐regulated under drought conditions in the tolerant genotypes ICC 4958 and JG 11+, compared to ICC 1882. Moreover, the expression level of *Ca_04564* was found to be lower in ICC 4958 and JG 11+ relative to JG 11 (Figure 6). Integration of transcriptome and proteome analysis revealed prominent expression variations in two genes (*Ca_04551* and *Ca_04564*) underlying the *“* *QTL‐hotspot”* region.

## 4 DISCUSSION

Drought stress is a complex trait and a major constraint to chickpea production worldwide. Identifying key genes regulating metabolic pathways underlying drought stress response mechanisms may facilitate the development of tolerant chickpea varieties at a faster pace (Roorkiwal et al., 2020). In addition to the identification of potential genes/proteins for drought stress response mechanism, it is important to develop climate‐smart drought‐tolerant varieties through genomics‐assisted breeding (Varshney, Bohra, Yu, et al., 2021; Varshney, Bohra, Roorkiwal, et al., 2021). This study aimed to better understand the drought stress response mechanism in chickpea through an integrated multi‐omics approach based on transcriptomics, proteomics, and metabolomics in contrasting drought‐responsive genotypes including a drought‐tolerant IL. The IL, JG 11+ is developed from the cross between tolerant genotypes ICC 4958 and JG 11, unlike a cross between tolerant and sensitive cultivars in routine breeding programs (Varshney, Song, et al., 2013). Hence, information on inherited genes/protein in JG 11+ from tolerant parents, ICC 4958 and JG 11, would be important. Overall, the results showed altered expression in several transcripts, proteins, and metabolites regulating defense signaling pathways under drought stress conditions. These results offer molecular insights into drought tolerance mechanisms in chickpea.

The RNA‐Seq data were analyzed for global gene expression patterns and the 32,217 gene loci identified in the current global gene expression analysis were considerably higher than the number of genes reported (28,269) in the chickpea genome by Varshney, Song, et al. (2013). This may be due to an incomplete (approximately, 74%) genome sequence available. Similar results were observed in earlier studies which showed higher number of genes than the number of genes annotated in the chickpea genome (Garg et al., 2016; Kudapa et al., 2018). Further, the comparison of reference‐guided assembly with the genome led to the identification of 4323 (13%) novel gene loci indicating the value of RNA‐Seq in the identification of novel genes. These observations confirm that this study provided a comprehensive resource of genome‐wide gene expression patterns under drought stress. The DEG analysis between control and stress conditions of different genotypes facilitated the identification of high number of DEGs in tolerant genotypes, which can be attributed to drought stress‐inducible genes. Similar results were observed in the transcriptome profiling of Iranian chickpea genotypes under drought stress conditions, where an increased number of DEGs was observed in Bivanij (tolerant) when compared to Hashem (sensitive) genotype (Mashaki et al., 2018). Furthermore, the difference in the number of DEGs between JG 11 and JG 11+ as well as ICC 4958 and JG 11 can be explained based on the introgression of the QTL‐hotspot region in JG 11+. For instance, the expression of genes in JG 11+ can be an outcome of the interaction between (a) trans‐regulatory factors from JG 11 and (b) cis‐regulatory elements in the QTL‐hotspot region. Also, the introgression of the *“* *QTL‐hotspot”* region may in turn influence background gene expression in JG 11+.

Further, the highest number of DEPs were also identified in tolerant genotypes, JG 11 followed by ICC 4958 supporting our results from transcriptome data. Proteomics data revealed proteins that were up‐ and downregulated under drought stress conditions, which might play a role in drought tolerance mechanism. For example, proteins such as annexin and vacuolar H+ ATPase V0 showed increased expression under stress conditions in the tolerant genotype, ICC 4958 compared to its control. Annexins are an evolutionarily conserved multigene family of calcium‐dependent phospholipid‐binding proteins and have relatively well‐characterized roles in stress resistance and plant development (Xu et al., 2016). The intrinsic activities of annexins include enzymatic activity, such as peroxidase and ATPase/GTPase activity, as well as Ca^2+^ channel activity (Gorecka et al., 2005). Overexpression of Arabidopsis *AnnAt1* conferred enhanced tolerance to drought stress (Konopka‐Postupolska et al., 2009). Similarly, the regulation of annexins was also identified in pearl millet roots under drought stress (Ghatak et al., 2016). Furthermore, to replenish water deficit conditions, roots also developed other mechanisms such as enhanced pumping of protons into vacuoles (Mohammadi et al., 2012). As a result, osmolytes and transmembrane water‐channel proteins such as vacuolar H+ ATPases are synthesized and stored in abundance in root tissue. In the present study, the transmembrane water‐channel protein, vacuolar H+ ATPase was upregulated in the stress sample of tolerant ICC 4958 compared to its control, indicating its significant role in defense mechanism.

Among the commonly expressed DEPs between ICC 4958 and JG 11+, LEA proteins constituted the most abundant category of protective proteins activated under drought stress conditions. Similar results have been reported earlier in Arabidopsis and South African resurrection plant *Craterostigma plantagineum* (Giarola et al., 2018). An increase in the expression levels of LEA and LEA‐like desiccation‐related protein PCC13‐62 is hypothesized to confer dehydration tolerance in JG 11+. The highly upregulated protein UDP‐d‐glucose/UDP‐d‐galactose 4‐epimerase, is an enzyme implicated to function in cell wall biosynthesis by modulating the monosaccharide pool accessible for pectin production (Reiter & Vanzin, 2001). Dehydration or consecutive periods of drought causes a decline in the membrane stability index and enhances the chances of membrane injury (Abid et al., 2018). Activation of the UDP‐d‐glucose/UDP‐d‐galactose 4‐epimerase under drought is predicted to contribute toward cell wall thickening in chickpea roots.

Pathogenesis‐related (PR) thaumatin family proteins were among the highly upregulated proteins inherited from JG 11 into JG 11+. The PR proteins in plants are known to be induced by various biotic and abiotic processes including drought (Wu et al., 2016). Callose synthase, a cell wall biosynthesis and metabolism protein, was reported to confer drought tolerance by enhancing the water‐holding capacity of the plants (Wang, Cai, et al., 2016) was induced under drought stress in JG 11 and JG 11+. Flavonoids represent a major group of plant secondary metabolites that serve as ROS scavengers and hence are important for plants exposed to adverse environmental conditions (Winkel‐Shirley, 2002). Anthocyanins form a major category of flavonoids in plants that are associated with diverse plant processes. UDP‐glucosyltransferases catalyze the last step in the anthocyanin biosynthesis pathway leading to a diverse array of anthocyanin molecules (Li et al., 2017). Interestingly, two proteins from the UDP‐glucosyltransferase family were found to be induced in JG 11 and JG 11+, which suggests that drought stress tolerance acquired by modulating anthocyanin accumulation is inherited from JG 11.

In addition, identified proteins common between all three tolerant genotypes suggested that these proteins can play a role in stress management. For example, dehydrins represent a category of LEA II proteins that are accumulated under numerous abiotic factors including drought, salinity, and low temperatures to prevent the aggregation, damage, and inactivation of proteins under stress conditions (Jing et al., 2016; Uçarlı et al., 2016). Upregulation of dehydrin DHN3‐like protein (*Ca_12999*) in tolerant chickpea genotypes suggests its role in regulating drought stress response mechanisms.

The integrated data analysis from transcriptomics and proteomics revealed significant differences in gene/protein expression patterns between contrasting chickpea genotypes under drought stress. A total of 60 DEPs identified in two or all the three tolerant genotypes did not show significant expression in ICC 1882 (sensitive) under drought stress conditions. Of these, a total of 10 proteins showed a correlation with corresponding DEGs from the transcriptome. Among these correlated proteins, desiccation‐related PCC13‐62‐like protein (DCP) showed significant upregulation. In plants, stress signals perceived by the cell induce cell wall remodeling to sustain flexibility and maintain ionic homeostasis (Hofmann, 2016). Differential expression of DCP in the present study indicates that tolerant genotypes undertook cell wall remodeling and membrane stabilization to protect them from the adverse effects of drought stress. Furthermore, the accumulation of the plant hormone abscisic acid (ABA) regulates drought stress responses by mediating stomatal closure and helping the plants to adapt to low water availability (Miao et al., 2018; Negin et al., 2019). The protein 65 kDa‐like (*Ca_04473*) known to be induced mainly by drought and ABA treatment was highly induced in the tolerant genotypes but did not display any differential expression in the sensitive genotype, ICC 1882 (Shi et al., 2015). From these results, it is evident that there is a good correlation between the transcriptome and the proteome datasets. However, such correlations are by no means universal due to posttranscriptional and posttranslational regulatory events in plant cells (Guerra et al., 2015; Gunawardana & Niranjan, 2013).

Furthermore, our results provided a comprehensive perspective on signaling pathways involved in chickpea drought stress response mechanisms, highlighting the importance of an integrated‐omics approach. Antibiotic biosynthesis, galactose metabolism, and isoflavonoid biosynthesis pathways were mainly enriched and are envisaged to be associated with drought stress response mechanisms in chickpea. Until recently, it was thought that gene clusters such as the synthesis of antibiotics are a common feature of microbes like fungi. However, studies in plants have discovered that gene clusters related to the synthesis of plant secondary metabolites/antibiotics are implicated in the synthesis of defense compounds (Chu et al., 2011). This may not be a common feature of all plant secondary metabolic gene clusters. In the present study, the pathway, synthesis of antibiotics has been enriched, indicating its role in defense mechanism under drought stress conditions. Similarly, galactose metabolism is another potential pathway reported to be induced under drought stress conditions (Xiao et al., 2019). Galactosyltransferase, a member of the galactose metabolism pathway, is known to be involved in cell wall biosynthesis and maintenance of endomembrane organization. It also minimizes cellular damage by limiting the ROS concentration in the cell and regulates stress‐related gene expression (Yang & Guo, 2018). Furthermore, malonylated isoflavones identified in the present study are the major forms of isoflavonoids known to alter isoflavone metabolic profiles. A recent study in soybean reported that modification of isoflavones (malonylated isoflavones—GmIMaT1 and GmIMaT3) plays important roles in plant growth and stress adaptation (Ahmad et al., 2017). Hence, understanding these differentially regulated pathways from the present study provided valuable insights into drought stress response mechanisms.

In addition to transcriptome and proteome analyses, a comprehensive metabolome profiling was performed in the present study to identify metabolites and their association with drought stress response mechanisms in chickpea. Increased amino acid levels enhance stress resilience in plants by influencing various mechanisms such as osmotic adjustments, ROS scavenging, regulation of intracellular pH levels, and so on. An increase in amino acid levels under drought stress was reported earlier in soybean (Silvente et al., 2012) and barley (Chmielewska et al., 2016). Accumulation of proline (Pro) was observed in the present study, which is known for its key role as an osmoprotectant in plants that are exposed to hyperosmotic stresses like drought (Ghatak et al., 2018). Proline protection against oxidative damage was reported in soybean (Das et al., 2017). In the present study, asparagine also showed higher accumulation under stress conditions in all the compared genotypes. Asparagine maintains nitrogen metabolism balance, which in turn improves the tolerance mechanism under stress conditions, and at the same time, it also plays a role in balancing the normal osmotic pressure (Oddy et al., 2020). Furthermore, an integration of transcriptomics, proteomics, and metabolomics analyses revealed an enhanced correlation of five metabolites with the enriched galactose metabolism pathway. Of these five metabolites, raffinose showed increased regulation under drought stress conditions in all the tolerant genotypes compared to the sensitive genotype ICC 1882. Raffinose acts as a protective compound by stabilizing membranes and mediating osmotic adjustment in plants (Taji et al., 2002).

To identify the role of the *“* *QTL‐hotspot”* in modulating drought tolerance in JG 11+, we compared the expression patterns of the candidate genes from the *“* *QTL‐hotspot”* at the transcriptome and proteome levels across four genotypes studied. Two genes, aldo/keto reductase family oxidoreductase (*Ca_04551*) and LRR/extensin (*Ca_04564*), common from both transcriptome and proteome analysis were prioritized. The aldo‐keto reductases are stress‐regulated genes reported to play a role in cellular responses to osmotic, electrophilic, and oxidative stresses. Recently, several aldo‐keto reductase genes from *Medicago truncatula* roots were found to be induced under drought stress (Yu et al., 2020). In Arabidopsis, the leucine‐rich repeat extensin (LRX) proteins, LRX1 and LRX2, were reported as major genes for cell wall formation during root hair development (Baumberger et al., 2003). In another study, the cell wall LRX 3/4/5 proteins were found to modulate plant growth and stress tolerance by transducing cell wall signals in Arabidopsis (Zhao et al., 2018). In summary, the present study demonstrated the potential of integrated multi‐omics analyses in providing a comprehensive overview of candidate genes and molecular mechanisms underpinning drought response in chickpea.

## AUTHOR CONTRIBUTIONS

**Himabindu Kudapa**: Data curation; Formal analysis; Investigation; Methodology; Validation; Writing – original draft; Writing – review & editing. **Arindam Ghatak**: Data curation; Formal analysis; Investigation; Methodology; Software; Validation; Visualization; Writing – original draft; Writing – review & editing. **Rutwik Barmukh**: Data curation; Formal analysis; Investigation; Methodology; Validation; Visualization; Writing – original draft; Writing – review & editing. **Palak Chaturvedi**: Data curation; Formal analysis; Investigation; Methodology; Software; Validation; Visualization; Writing – original draft; Writing – review & editing. **Aamir Khan**: Data curation; Formal analysis; Investigation; Software; Validation; Writing – review & editing. **Sandip Kale**: Data curation; Formal analysis; Investigation; Software; Visualization; Writing – review & editing. **Lena Fragner**: Methodology; Resources; Writing – review & editing. **Annapurna Chitikineni**: Methodology; Resources; Writing – review & editing. **Wolfram Weckwerth**: Investigation; Resources; Visualization; Writing – original draft; Writing – review & editing. **Rajeev K. Varshney**: Conceptualization; Funding acquisition; Methodology; Project administration; Resources; Supervision; Visualization; Writing – original draft; Writing – review & editing.

## CONFLICT OF INTEREST STATEMENT

The authors declare no conflicts of interest.

## Supporting information

Supplemental Table S8: List of metabolites mapped to different pathways involved in chickpea drought stress response mechanism

Supplemental Table S1: Details on RNA‐seq data generated from control and stress root tissues of four chickpea genotypes

Supplemental Table S2: Expression values (FPKMs ≥ 1) on a per gene basis for all four chickpea genotypes under control and drought stress conditions

Supplemental Table S3: The number of transcriptionally active genes and distribution of expressed genes in eight chickpea samples into low/moderate and high expression categories

Supplemental Table S4: List of differentially expressed genes (DEGs), their expression (raw FPKM) and annotation across the four chickpea genotypes

Supplemental Table S5: List of differentially expressed genes (DEGs) for different combinations of genotypes under drought stress conditions

Supplemental Table S6: List of proteins, their expression and annotation across four chickpea genotypes

Supplemental Table S7: List of pathways involved in drought stress response mechanism in chickpea roots

Supplemental Figure S1: Integrated multi‐omics analysis to understand chickpea drought stress response mechanism.
Supplemental Figure S2: Expression profiles of differentially expressed genes (DEGs).
Supplemental Figure S3: Global proteome regulation and differentially expressed proteins.
Supplemental Figure S4: Expression profiles of differentially expressed proteins (DEPs).
Supplemental Figure S5: Graphical representation of major pathways identified from the correlated analysis of DEGs and DEPs.
Supplemental Figure S6: Principal component analysis of all measured metabolites under control and drought stress condition (performed using COVAIN software; Sun and Weckwerth, 2012).

## Data Availability

All transcriptomics data generated have been deposited into National Center for Biotechnology Information (NCBI) Sequence Read Archive (SRA) database under the BioProject ID: PRJNA738320. MS/MS Spectra dataset is available in the PRIDE repository. Project accession: PXD027033.
