# Supplementary material for: Integrated multi‐omics analysis reveals drought stress response mechanism in chickpea (Cicer arietinum L.)
Source: Plant Genome. 2023 May 10;17(1):e20337. doi: 10.1002/tpg2.20337 (PMC12806944; doi:10.1002/tpg2.20337)
Supplement: Supplementary file 1 [file TPG2-17-e20337-s009.docx]

**Integrated multi-omics analysis reveals drought stress response mechanism in chickpea (*Cicer arietinum* L.)**

Himabindu Kudapa^1,$^, Arindam Ghatak^2,$^, Rutwik Barmukh^1,$^,

Palak Chaturvedi^2^, Aamir Khan^1^, Sandip Kale^3^, Lena Fragner^2^,

Annapurna Chitikineni^1^, Wolfram Weckwerth^2,4^, Rajeev K Varshney^1,5,*^

^1^Center of Excellence in Genomics & Systems Biology, International Crops Research Institute for the Semi-Arid Tropics (ICRISAT), Patancheru, Hyderabad, India

^2^Molecular Systems Biology Lab (MOSYS), Department of Functional and Evolutionary Ecology, University of Vienna, Austria

^3^The Leibniz-Institute of Plant Genetics and Crop Plant Research (IPK), Corrensstr. 3,06466 Seeland, OT Gatersleben, Germany

^4^Vienna Metabolomics Centre (VIME), University of Vienna, Austria

^5^Murdoch’s Centre for Crop & Food Innovation, State Agricultural Biotechnology Centre, Food Futures Institute, Murdoch University, Murdoch, Western Australia, Australia

^$^Authors contributed equally

^*^Correspondence: [rajeev.varshney@murdoch.edu.au](mailto:rajeev.varshney@murdoch.edu.au)

**Supplemental Figures**

**Supplemental Figure S1: Integrated multi-omics analysis to understand chickpea drought stress response mechanism.**

**Supplemental Figure S2:** **Expression profiles of differentially expressed genes (DEGs)**. Heatmaps show the expression patterns of DEGs in the two tolerant genotypes and introgression line. The genotype combinations are (a) ICC 4958 and JG 11+, (b) JG 11 and JG 11+, and (c) ICC 4958, JG 11 and JG 11+. The colour scale represents normalized expression values.

**Supplemental Figure S3:** **Global proteome regulation and differentially expressed proteins**. (a) Venn diagram demonstrates proteome regulations of all the four chickpea genotypes under control and stress condition. (b) Genotype specific DEPs (up-, down- regulated and non-significant proteins) are represented using volcano plots.

**Supplemental Figure S4:** **Expression profiles of differentially expressed proteins (DEPs)**. Heatmaps show the expression patterns of DEPs in the two tolerant genotypes and introgression line. The genotype combinations are (a) ICC 4958 and JG 11+, (b) JG 11 and JG 11+, and (c) ICC 4958, JG 11 and JG 11+. The colour scale represents normalized expression values.

**Supplemental Figure S5: Graphical representation of major pathways identified from the correlated analysis of DEGs and DEPs**. Radial bars represent the number of genes in each pathway and the numbers designated against each pathway on the chart represents the number of enzymes.

**Supplemental Figure S6: Principal component analysis of all measured metabolites under control and drought stress condition** (performed using COVAIN software; Sun and Weckwerth, 2012). The two principal components showed clear demarcation in the metabolites identified between drought stress and control root samples of all four chickpea genotypes.
